# Can Diabetes Drugs like Ozempic Tackle the Mental Health Crisis?

**DOI:** 10.1021/acscentsci.4c01250

**Published:** 2024-08-12

**Authors:** Elizabeth Hlavinka

For Kelli Coviello, injections of Wegovy didn’t just satiate her appetite and help her lose weight. They lowered her blood pressure, improved her mood, and helped her think clearly.

Coviello, a principal’s assistant at an elementary school in Massachusetts, would typically get home from work, eat something, and go to bed. A month after starting Wegovy, she has the energy to help a friend campaign for local government, prepare her son’s dinner, and go on a walk in the evening instead. Although she is already on antianxiety medication, Wegovy (semaglutide) helped alleviate her anxiety and depression like nothing else had.

“I feel like me again,” Coviello says.

Semaglutide, the same compound that’s in Ozempic, is part of a class of medications known as glucagon-like peptide 1 (GLP-1) receptor agonists. These drugs mimic GLP-1 hormones naturally released in the body when eating.

Scientists have long known that these hormones prompt the body to make insulin, producing feelings of satiation. On this basis, the U.S. Food and Drug Administration approved Ozempic for type 2 diabetes in 2017 and Wegovy for weight loss in 2021.

Because these hormonal pathways can be tied into numerous bodily processes, researchers are now trying to study what else GLP-1 analogs could potentially treat. Early signs indicate they may have a role to play in treating a mass crisis: mental health.

Earlier this year, data from about 4 million patients published by Epic Research, a data science arm of the medical record company Epic Systems, showed that semaglutide was linked with reductions in the prevalence of anxiety and depression diagnoses. And for patients with diabetes, nearly every GLP-1 receptor agonist on the market was associated with reduced diagnoses of the two conditions.

Clinical trials are underway to test these preliminary signals and determine whether GLP-1 analogs can reduce symptoms for people with depression, schizophrenia, addiction, and bipolar disorder—along with neurological conditions like Alzheimer’s disease and Parkinson’s disease. Why these drugs might be able to treat many of these neurological and psychiatric conditions is unclear, but finding out how exactly they work could help us better understand the body-and-mind connection and treat millions of people worldwide.

“For some people, these meds do change everything, including mood, when antidepressants haven’t really done that much,” says Karen S. Greenberg, a psychiatrist affiliated with the Beth Israel Deaconess Medical Center.

## Mind meets metabolism

Scientists’ hypothesis that GLP-1 drugs have a role to play in mental health is in part fueled by decades of research that shows that metabolic health and mental health are closely intertwined.

For example, type 2 diabetes is more prevalent in people with bipolar disorder, schizophrenia, and depression than in people without those conditions. Moreover, research has shown that there are genetic links between type 2 diabetes and psychiatric disorders. In one 2024 study, high levels of glucose and triglycerides and low levels of high-density lipoprotein—which are usually signs of worse metabolic health—were associated with future risk of depression and anxiety (*JAMA Network Open*, DOI: 10.1001/jamanetworkopen.2024.4525).

Metabolic and psychiatric systems are not just indirectly linked, though. Evidence shows that a treatment for one can influence the other. For instance, many antidepressant medications cause weight gain, according to the CDC. And in a 2022 study of people with treatment-resistant bipolar depression, participants’ depression and functioning improved upon the reversal of insulin resistance—a metabolic condition that is more common in people with bipolar disorder.

Molecular-level observations also add to the evidence that GLP-1 is active in the brain. Naturally occurring GLP-1 activates the hypothalamus, a part of the brain that controls aspects of appetite and food intake, says Zhiping Pang, a neuroscientist at Robert Wood Johnson Medical School and the Child Health Institute of New Jersey. But Pang adds that the hormone also acts on parts of the brain that control seemingly disparate mental processes, such as the mesolimbic—or reward—system and the hippocampus, a learning and memory center that many neurological and psychiatric disorders affect.

These physiological observations, in addition to person-level studies, suggest that GLP-1 drugs could represent an entirely new pathway for treating mental health conditions.

**Figure:**
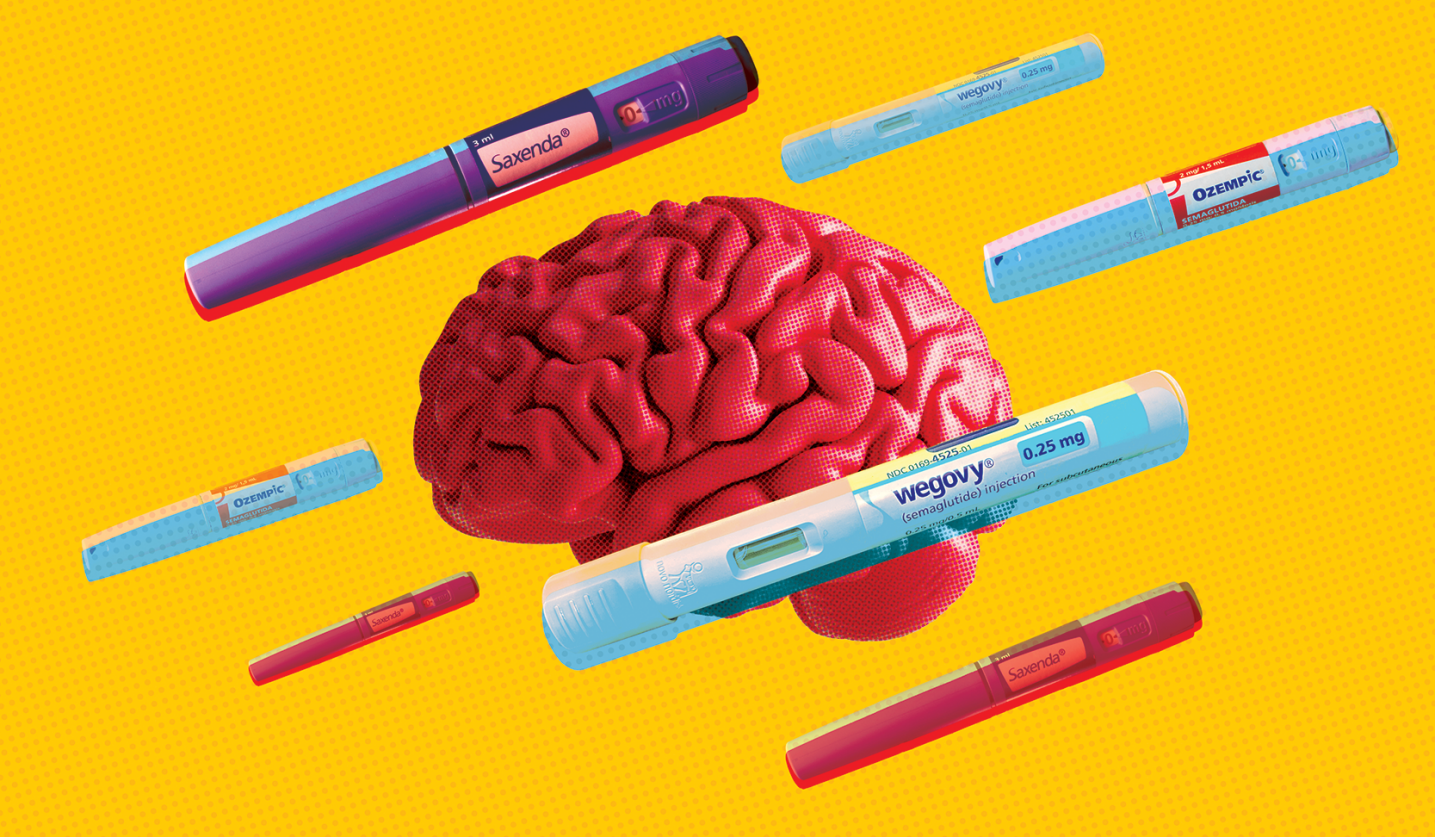
Credit: Madeline Monroe/C&EN/Shutterstock.

## Making drugs that stick around

Despite all their activity in the body and brain, naturally occurring GLP-1 hormones don’t make good drugs. Their half-lives in the body are relatively short, so they don’t stick around long enough to have a lasting effect. In designing synthetic GLP-1 drugs, pharmaceutical companies outfit the molecules with the capacity to evade breakdown by enzymes. In other words, the compounds stay in the body longer and have a greater effect.

Naturally occurring GLP-1 peptides contain 30 or 31 amino acids and are broken down by dipeptidyl peptidase 4 (DPP-4). Synthetic GLP-1 drugs each have their own modification that allows them to slip by DPP-4 or survive longer in the blood. Semaglutide, for example, highly resembles naturally occurring GLP-1, but Novo Nordisk scientists substituted an amino acid at the second position. This swap—an isobutyric acid in place of an alanine—prevents the drug from being degraded as quickly, says Richard DiMarchi, a chemistry professor at Indiana University Bloomington who was a group vice president at Eli Lilly and Company and later at Novo Nordisk. (DiMarchi also sold two of his diabetes-related start-ups to Novo Nordisk.)

Scientists also installed a fatty acid onto semaglutide with a carboxyl group dangling off the end. The fatty acid is key because it makes the drug bind more strongly to albumin in the blood, meaning it sticks around longer before getting cleared, DiMarchi says.

That interaction “allows you to have a once-a-week administration because [the drug] circulates for a longer time,” he says.

On the other hand, the type 2 diabetes drug exenatide was created a little differently. Approved by the FDA in 2005, long before semaglutide, it is a mimic of a 39-amino acid peptide called exendin-4, which was originally isolated from a type of lizard known as the Gila monster. Later, scientists realized the peptide suppressed appetite, and they designed a synthetic version of it called exenatide. In order to prevent degradation by DPP-4, scientists swapped the alanine at the inactivation site with a glycine.

The extra 9 amino acids on exenatide’s C-terminus help increase the drug’s solubility and prevent the drug molecules from clumping up in the body, DiMarchi says.

In addition to standing up to DPP-4’s breakdown action better than analogous human hormones, some of the first-generation GLP-1 drugs, like exenatide, have also been shown to cross the blood-brain barrier in rodents. It is unclear whether newer molecules like semaglutide, which are larger and cause more weight loss than some of the earlier iterations, can cross the blood-brain barrier too. If they can, that could strengthen the case for testing whether they have a role to play in mental health treatment.

But even if these compounds are not directly crossing the blood-brain barrier, scientists still think they act on the brain through some secondary pathway. For instance, they could be passing through circumventricular organs—parts of the brain where there are gaps in the blood-brain barrier. Or they may be acting on the vagus nerve, which connects the gut to the brain.

Understanding whether these drugs are physically crossing into the brain or having an effect by some secondary mechanism is important because drugs that cross the blood-brain barrier can be more effective but also introduce safety risks, DiMarchi says. “It is the double-edged sword that is a common challenge in the development of brain-active drugs.”

**Figure:**
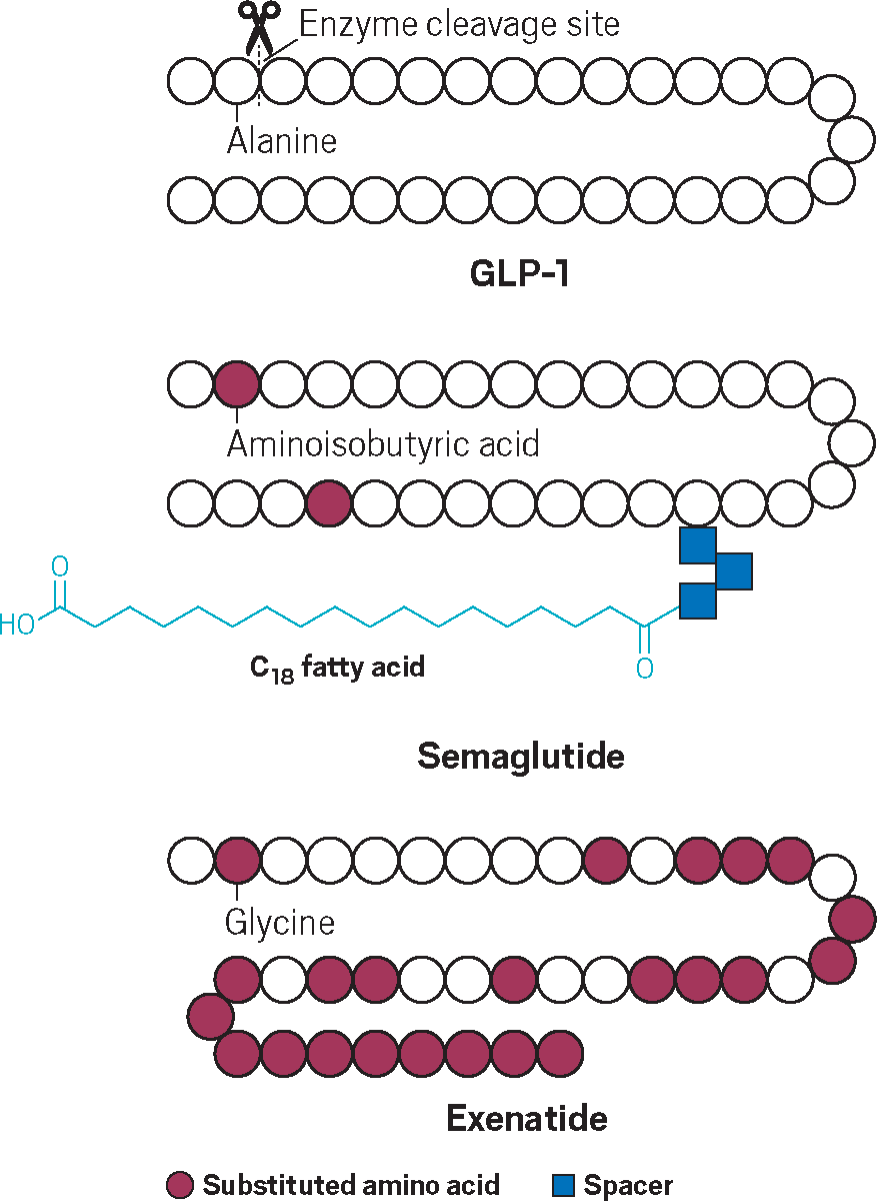
Semaglutide and exenatide circulate in the body longer than natural glucagon-like peptide 1 hormones thanks to a substituted amino acid at the second position of the drugs’ peptide chains. Those amino acids prevent enzymes from cutting and deactivating the drugs. Credit: Adapted from *Adv. Drug Delivery Rev.*

## The case for testing GLP-1s on the mind

Efforts to understand GLP-1’s role in diabetes have existed since the 1980s. Around that time, researchers first sequenced glucagon, a hormone that keeps blood sugar from getting too low. Scientists developed GLP-1 receptor agonists to inhibit the release of glucagon, but efforts to understand GLP-1’s role in mental health are more recent.

In 2015, before the FDA approved synthetic GLP-1s for weight loss and researchers linked them to cardiovascular, kidney, and lung function, Rodrigo Mansur, a psychiatrist at the University of Toronto, hypothesized that GLP-1 drugs might help treat mental illness.

But colleagues met Mansur with skepticism, he says. “Most of the reactions we got were basically, ‘Why are you testing these antidiabetes [drugs] for psychiatry? That doesn’t make any sense,’ ” he recalls.

Mansur is focused on finding a treatment that targets the cognitive elements of depression, such as impaired learning or memory and reduced attention or concentration. Because cognitive decline and impaired memory are associated with insulin resistance, he decided to test whether GLP-1 drugs could be a potential treatment.

He found that the GLP-1 drug liraglutide improved cognition among people with depression and bipolar disorder. Although there was no comparator arm in his 2016 study and all patients received the drug, the more weight that patients lost on liraglutide, the more their cognition improved (*J. Affective Disord.*, DOI: 10.1016/j.jad.2016.09.056). Mansur is conducting a larger trial with a placebo comparator group to further examine liraglutide’s effect on cognition in people with depression.

“Most of the standard treatments for depression, they tend to be helpful for the more emotional aspects of depression like mood, but they don’t help the cognition as much,” Mansur says. “So this is an area where there is a need to develop new treatments.”

Mahavir Agarwal, a psychiatrist who coleads the Mental Health and Metabolism Clinic at the Centre for Addiction and Mental Health, says GLP-1 drugs are worth investigating if only for antipsychotic side effect management in people with psychiatric conditions.

Many agree that civil rights laws should protect against weight-based discrimination, but higher weight is still highly stigmatized, which harms people’s mental health. Agarwal says many of his patients stop taking antipsychotics because of possible weight gain, and their mental health conditions get worse.

Plus, many patients in his clinic develop cardiovascular disease or diabetes as adverse effects of their medication in addition to their psychiatric conditions. If GLP-1 drugs could reduce these side effects of antipsychotics, they could help some patients stay on their medication and reduce the prevalence of those accompanying diseases.

“That in itself would be a huge victory,” Agarwal says.

## How GLP-1 drugs might work

Scientists have proposed several theories to explain why GLP-1 drugs act on the brain. One involves the neurocircuitry of addiction.

In mouse studies, administering a GLP-1 analog has been shown to reduce alcohol and cocaine use. Specifically, exenatide worked by reducing alcohol’s and cocaine’s abilities to release dopamine, a neurotransmitter associated with desire and motivation, in the brain (*Br. J. Pharmacol*. 2021, DOI: 10.1111/bph.15677).

The mental effects of GLP-1 analogs show up in areas of life beyond addiction. Coviello, the principal’s assistant in Massachusetts, describes how semaglutide helps give her more drive. Before she started taking the medication, she felt overwhelmed by a sense of apathy that kept her from exercising and staying active, she says.

“Things just balloon up to a point that you feel like nothing’s working, and then you feel like, ‘OK, now I’m so far gone, what’s the point?’ ” Coviello says.

For her, taking Wegovy is hardly about the weight loss anymore. “I care again,” she says.

This newfound vigor, along with anecdotal reports of people curbing other behaviors like biting their nails, indicates there is something more going on than just these drugs’ triggering satiety or slowing down gut motility, says neuroscientist Miriam Bocarsly, who studies the neural circuitry involved with eating at Rutgers New Jersey Medical School. “I think something is changing in the reward pathway in the brain.”

GLP-1 drugs like semaglutide stimulate the production of insulin, which can cross the blood-brain barrier. That stimulation might be causing a cascade of other chemical changes that ultimately affects mood. Bocarsly adds that research in her lab found that increasing insulin in the reward center of the brain in mice also increased dopamine, and dopamine dysregulation is present in Parkinson’s disease, schizophrenia, and bipolar disorder.

“We know that these drugs are affecting insulin in the periphery in good ways, and we know that insulin goes into the brain,” Bocarsly says. “This is all very hand-wavy theory right now, but could it be the insulin that’s having some of these effects in the brain?”

It is clear that insulin dysregulation affects the brain, as evidenced by certain neurodegenerative diseases. Some unofficially call Alzheimer’s disease type 3 diabetes, for example, because issues with insulin signaling are tied to the accumulation of peptides characteristic of Alzheimer’s.

Olivier Rascol, a neurologist at Toulouse University Hospital, presents another hypothesis, with implications for neurodegenerative diseases: GLP-1 drugs could be reducing inflammation. In April, Rascol’s team published research in the *New England Journal of Medicine* that found the GLP-1 drug lixisenatide reduced movement-related symptoms in people with early stage Parkinson’s (2024, DOI: 10.1056/NEJMoa2312323). Increased inflammation is prevalent in people with depression and conditions like Parkinson’s, Rascol says.

While Rascol’s leading hypothesis is that GLP-1 drugs are working to reduce inflammation, he acknowledges that the reductions in Parkinson’s symptoms in his study could also be explained by something else. For example, GLP-1 drugs could somehow enhance the effect of levodopa, a treatment for Parkinson’s that all patients in the study were on. “There are all these different potential mechanisms of action, which are all basically just that they help neurons to survive,” Rascol says.

Preliminary results from studies on GLP-1 analogs and mental health disorders could be forthcoming as soon as next year. But scientists have yet to discover whether they reduce neuroinflammation, trigger some neural connection that works on multiple systems, or have some other unknown mechanism.

Time will tell whether GLP-1 drugs become something more than a treatment for type 2 diabetes and a means to lose weight. Meanwhile, millions of people with mental illnesses await the results.

If GLP-1 drugs can give those living with mental illnesses and neurodegenerative diseases reprieve, “I would compare it to some of the major breakthroughs of the history of medicine,” Rascol says. “In Parkinson’s disease, that would be the discovery of levodopa, for example...or the discovery of insulin, the discovery of antibiotics.”

## Elizabeth Hlavinka is a freelance contributor to

Chemical & Engineering News, *the independent news outlet of the American Chemical Society.*

